# EZH1 Is Associated with TCP-Induced Bone Regeneration through Macrophage Polarization

**DOI:** 10.1155/2018/6310560

**Published:** 2018-08-30

**Authors:** Xiaoshi Jia, Hudi Xu, Richard J. Miron, Chengcheng Yin, Xiaoxin Zhang, Min Wu, Yufeng Zhang

**Affiliations:** ^1^The State Key Laboratory Breeding Base of Basic Science of Stomatology (Hubei-MOST) and Key Laboratory of Oral Biomedicine Ministry of Education, Wuhan University, Wuhan, China; ^2^Department of Periodontology, University of Bern, Bern, Switzerland; ^3^Hubei Key Laboratory of Cell Homeostasis, Hubei Key Laboratory of Developmentally Originated Disease, Department of Biochemistry and Molecular Biology, College of Life Sciences, Wuhan University, Wuhan, Hubei 430072, China; ^4^Department of Oral Implantology, Wuhan University, Wuhan, Hubei 430072, China

## Abstract

Macrophages have been found to regulate the effects of biomaterials throughout the entire tissue repair process as an antigen-presenting cell. As a well-defined osteoconductive biomaterial for bone defect regeneration, tricalcium phosphate (TCP) has been found to facilitate a favourable osteoimmunomodulatory response that can shift macrophage polarization towards the M2 phenotype. In the present study, our group discovered that a histone methyltransferase enhancer of zeste1 (EZH1) was drastically downregulated in Thp1 cells stimulated by TCP, indicating that EZH1 may participate in the macrophage phenotype shifting. Furthermore, the NF-*κ*B pathway in macrophages was significantly downregulated through stimulation of TCP, suggesting a potential interaction between EZH1 and the NF-*κ*B pathway. Utilizing gene knock-down therapy in macrophages, it was found that depletion of EZH1 induced M2 macrophage polarization but did not downregulate NF-*κ*B. When the NF-*κ*B pathway was inhibited, the expression of EZH1 was significantly downregulated, suggesting that the inhibition of EZH1 may be regulated by the NF-*κ*B pathway. These novel findings provide valuable insights into a potential gene target system that controls M2 macrophage polarization which ultimately favours a microenvironment suitable for bone repair.

## 1. Introduction

As a type of antigen-presenting cell, macrophages have been found to regulate implanted biological materials during the tissue repair process [1]. The strong plasticity of macrophages enables their dual function that favours either tissue inflammation or tissue repair, which have made macrophages an ideal research target in the field of tissue engineering [2, 3]. With changes to the surrounding microenvironment, macrophages can polarize into two phenotypes, either M1 (tissue inflammation) or M2 (tissue repair) macrophages. It is generally accepted that the M1 macrophage produces a number of proinflammatory cytokines including IL-6, IL-12, and TNF-alpha, thereby reducing osteogenic differentiation [4]. On the contrary, M2 macrophages are responsible for tissue repair macrophage and hence secrete pro-wound-healing cytokines and growth factors including TGF-*β*, VEGF, and IFG-1 to promote immunoregulation and tissue repair [5]. Therefore, balancing the microenvironment and macrophage polarization between M1 and M2 phenotypes is critical for proper biomaterial implant integration.

As a well-defined osteoconductive biomaterial for bone regeneration, tricalcium phosphate (TCP) has broad clinical applications [6, 7]. Because of the good absorbability and osteoconduction characteristics of TCP, it enables bone remodelling to occur at the material interface [8–10]. Naturally, however, it is important to understand that prior to bone formation around any biomaterial, immune cells (namely, macrophages) surround the surface of the biomaterial and create a microenvironment that favours either future bone regeneration or fibrous encapsulation [3].

There have been inconsistencies in the literature defining the role of macrophages with respect to their contribution to osteogenesis around TCP bone particles. In a study conducted by Chen et al., it was revealed that macrophage polarization was driven towards the M1 extreme when cobalt was incorporated into *β*-tricalcium phosphate and this negatively impacted the ability for bone-forming osteoblasts to contribute to new bone formation [11]. Similarly, a study by Tai et al. found that TCP particles promoted macrophage expression of CD86, an M1 surface molecule in macrophages [12]. In contrast, however, a study by Chen et al. found that TCP extracts facilitated a favourable osteoimmunomodulatory response that promoted M2 macrophage polarization including the release of bone morphogenetic protein-2 (BMP2), one of the best-known inducers of osteoblast differentiation of bone marrow stromal cells (BMSCs) [13, 14].

Currently, numerous studies have shown that histone methylation modification plays an important role in the polarization of macrophages [15–17]. Previously, our group revealed that EZH1 can form a nonclassical complex with SUZ12 and UTX, leading to the assembly of RNA polII to the target genes of NF-kappa B [18]. NF-kappa B is a key pathway that mediates macrophage polarizations towards the M1 phenotype, but whether it can regulate EZH1 remains unknown. Furthermore, the direct role of EZH1 during macrophage polarization remains unclear [19, 20].

In the present study, we aimed to investigate the potential role of macrophages during the bone regeneration process in the femurs of rats implanted with TCP. It was revealed that TCP significantly suppresses the activation of the NF-kappa B pathway and causes a decrease in EZH1 expression. The reduction of EZH1 led to lower expression of M1 markers resulting in a lowered release of proinflammatory cytokines. As a result, it was found that this local microenvironment favoured bone regeneration. We then set to characterize the role of EZH1 in the regulation of macrophage polarization and further aimed to clarify the interaction between EZH1 and NF-kappa B during the bone repair process.

## 2. Materials and Methods

### 2.1. In Vivo Studies by Using the Femur Defect Model

The tricalcium phosphate utilized in this study was purchased from Olympus Terumo Biomaterials. Six mature Wistar rats whose body weight ranged from 185 to 235 grams were utilized for the in vivo component of this study. All rats utilized in this study were handled in accordance with the policies of the Ethics Committee for Animal Research, Wuhan University, China. Proper sterile conditions and minimally invasive surgical procedures were utilized throughout the entire surgery and approved by the Ethics Committee for Animal Use of the Institute of Biomedical Sciences, under protocol number 134/2012. The animals and surgical protocols were performed according to our previous studies [21]. Briefly, the femoral diaphysis was orientated by a direct incision towards the fascia lata. A bone defect that reached the medullary canal was made by a diamond-tipped drill bit at 4000 rpm irrigated by physiological serum. Then the defect was filled with sterilized TCP. Afterwards, the wound was sutured closed in 2 layers. After one day, 3 rats were sacrificed using sodium pentobarbital (200 mg/kg, ip). After 8 weeks, the remaining three rats were sacrificed.

### 2.2. Hematoxylin-Eosin Staining

After fixing in 4% formaldehyde for 48 hours, femoral samples were submersed in 10% EDTA, which was changed every three days for 3 weeks to decalcify the samples. Then the samples were embedded in paraffin. Samples were cut in 5 *μ*m thick sections and each slide was prepared with two sections. The sections were stained using hematoxylin-eosin stain (Sigma).

### 2.3. Cell Culture and Extract Preparation

Thp1 cells were cultured at 37°C with 5% carbon dioxide in HyClone RPMI 1640 medium modified (Thermo Fisher Scientific Inc.), 10% fetal bovine serum (FBS) (Gibco, Life Technologies Corporation), and 100 mg/mL streptomycin (HyClone). TCP was sterilized and placed into 24-well plates (100 mg/well) using a small spatula and then into cultures with 500 *μ*L/well of Thp1 culture medium. After 24 h of incubation, the culture medium was collected as the TCP extract.

Thp1 was first induced to macrophages by culture with 100 ng/mL of phorbol myristate acetate (PMA) (Sigma) at a density of 1 × 10^6^ cells/mL for 48 h. Then the culture medium was changed to culture medium containing TCP extract, 500 ng/mL lipopolysaccharide (LPS) (PeproTech), and 20 ng/mL human interleukin-4 (IL-4) (PeproTech). After incubation for 1 h, the supernatants of Thp1 cells cultured with TCP extract were collected.

Human bone marrow stem cells (HBMSCs) were obtained from patients undergoing iliac bone graft with informed written consent. The procedure was approved by the Ethics Committee at Wuhan University, China. HBMSCs were cultured in a-MEM medium (Thermo Fisher Scientific Inc.) at 37°C with 5% carbon dioxide.

### 2.4. Gene Silence and Osteogenic Induction

Gene silence in Thp1 was performed as Lipofectamine 3000 reagent protocol (Thermo Fisher Scientific). The target sequence for siRNA was 5′-CCAAAGUGGUCAUGGUGAATT-3′.

Osteogenic-inducing media were comprised of a-MEM with 10% FBS, 100 U/mL penicillin, 100 mg/mL streptomycin, 10 nM dexamethasone, 10 mM beta-glycerophosphate, 50 *μ*g/mL L-ascorbic acid, and TCP extract, Thp1 supernatants.

### 2.5. Alizarin Red Staining and Alkaline Phosphatase Staining

HBMSCs were first washed with PBS three times and then fixed in 4% formaldehyde for 15 min after being induced with osteogenic differentiation media for 14 days. Then samples were stained with 0.1% alizarin red solution (pH 4.2) at room temperature for 1 h. Alkaline phosphatase (ALP) staining was performed according to the manufacturer's protocol (ALP; Beyotime) after HBMSCs were cultured for 7 days. Quantification of the areas of alizarin red staining and ALP staining was measured by ImageJ software [22]. Stained areas were designated by normalizing the threshold and then analysing the size of the stained areas.

### 2.6. Protein Extraction and Western Blotting

EZH1 silenced and normal Thp1 cells were treated with TCP extract for 1 h. Then cells were collected with RIPA, mixed with loading buffer, and then heated for 10 min at 95°C to denature total protein for Western blotting. The antibodies against EZH1 (ABclonal, A5818), P65 (Boster, BA0610-2), and P-P65 (Cell Signaling Technology Inc.) were purchased from the indicated companies.

### 2.7. Reverse Transcription and Quantitative PCR

Thp1 cells were treated with PMA for 48 h, and then the culture medium was replaced with culture medium containing TCP extract, LPS, and IL-4. After incubation for 1 h, the supernatants of Thp1 were collected. Then HBMSC cells were treated with culture medium including the supernatants from the Thp1, LPS, IL-4, and TCP extract which contained osteogenic differentiation medium for 14 days. Thp1 cells were treated with culture medium, LPS, IL-4, and TCP extract for 2 h to evaluate gene expression for macrophage polarization. Total RNA was extracted with TRIzol reagent (TriPure Isolation Reagent, Roche Applied Science, Germany) following the manufacturer's protocol. About 1 *μ*g of total RNA was used in reverse transcription with PrimeScript RT-PCR Kit (TaKaRa, Japan) according to the manufacturer's protocol. The primer sequences used in Q-PCR are shown in Table 1.

### 2.8. Statistical Analysis

All data analysis was performed using GraphPad Prism software (San Diego, CA). The significance was set at *p* < 0.05. Data was calculated as a mean, and the statistical significance of differences between different groups was examined by one-way ANOVA and *t*-test. GAPDH was used as housekeeping gene. ^∗^*p* < 0.05, ^∗∗^*p* < 0.01, ^∗∗∗^*p* < 0.001, and ^∗∗∗∗^*p* < 0.0001.

## 3. Results

### 3.1. TCP Scaffolds Promote Bone Regeneration and BMSC Osteogenic Differentiation

**Figure 1:**
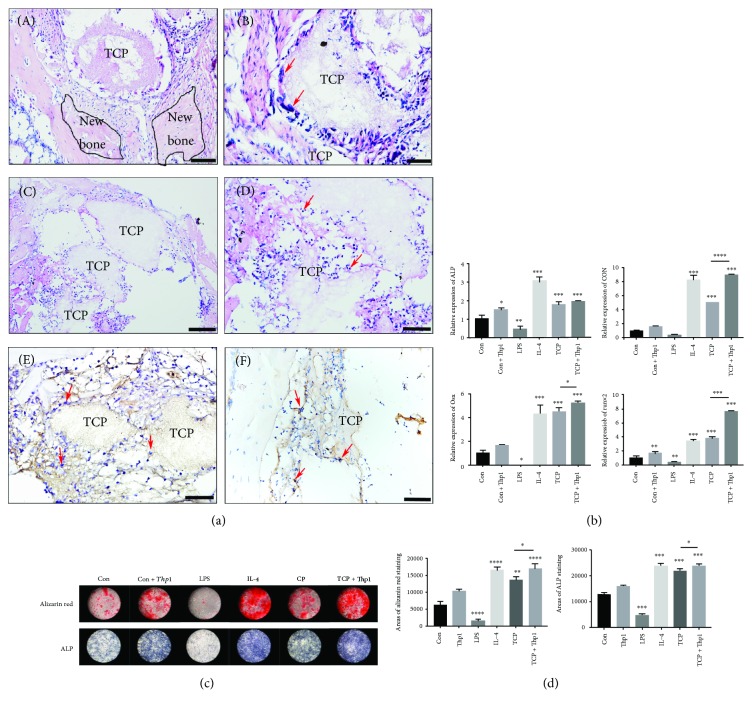
Osteogenic effect and gathering of macrophages at the healing defects filled with TCP. (a) (A–D) Representative sections of hematoxylin-eosin staining. Macrophages are indicated by red arrows. (E) Representative sections of immunohistochemistry staining with F4/80 antibody. Macrophages are indicated by red arrows. (F) Representative sections of immunohistochemistry staining with CD206 antibody. M2 macrophages are indicated by red arrows. (A,C) Scale bar = 50 *μ*m; (B, D, E, and F) scale bar = 20 *μ*m. (b) Relative mRNA expression of ALP, OCN, OSX, and Runx2 of HBMSC stimulated by control medium, supernatant of control Thp1, LPS, IL-4, and TCP extract and supernatant of TCP-induced Thp1. (c) Alizarin red staining, ALP staining, and quantification of HBMSC stimulated by control medium, supernatant of control Thp1, LPS, IL-4, and TCP extract and supernatant of TCP-induced Thp1. (d) Quantification of the areas of alizarin red staining and ALP staining was measured by ImageJ software. ^∗^*p* < 0.05, ^∗∗^*p* < 0.01, ^∗∗∗^*p* < 0.001, and ^∗∗∗∗^*p* < 0.0001.

To confirm the ability of TCP scaffolds during bone regeneration and to investigate the potential bone-promoting mechanism, TCP scaffolds were implanted in the femur of rats and sacrificed after 1 day and 8 weeks. After 8 weeks, new bone formation around TCP scaffolds was visible by H&E staining (Figures 1(c) and 1(d)). More in light with the early events taking place during TCP/biomaterial, it was found that after 1 day in vivo, macrophages gathered around the TCP particles prior to bone regeneration (Figures 1(a), 1(b), 1(e), and 1(f)).

Experiments from our in vitro data further demonstrated that TCP stimulates BMSC osteogenic differentiation. Firstly, since TCP is mineralized and can be stained with alizarin red (ARS), we utilized TCP extract for all the in vitro experiments. As illustrated in Figures 1(b)–1(d), the extract of TCP scaffolds significantly upregulated osteogenic differentiation markers when compared to the control group (Figures 1(b)–1(d)). Furthermore, the impact of inflammation can be markedly seen through the positive role of anti-inflammatory mediator IL-4, whereas a negative role was observed in the proinflammatory mediators which included LPS during osteoblast differentiation. Compared to the control group, stimulation of Thp1 with the supernatant of the TCP extract induced significantly better osteogenic differentiation (Figure 1). We therefore began to focus on the role of macrophage induced via TCP during BMSC osteogenic differentiation to investigate a potential mechanism for this action.

### 3.2. TCP Scaffolds Facilitate M2 Macrophage Polarization

**Figure 2:**
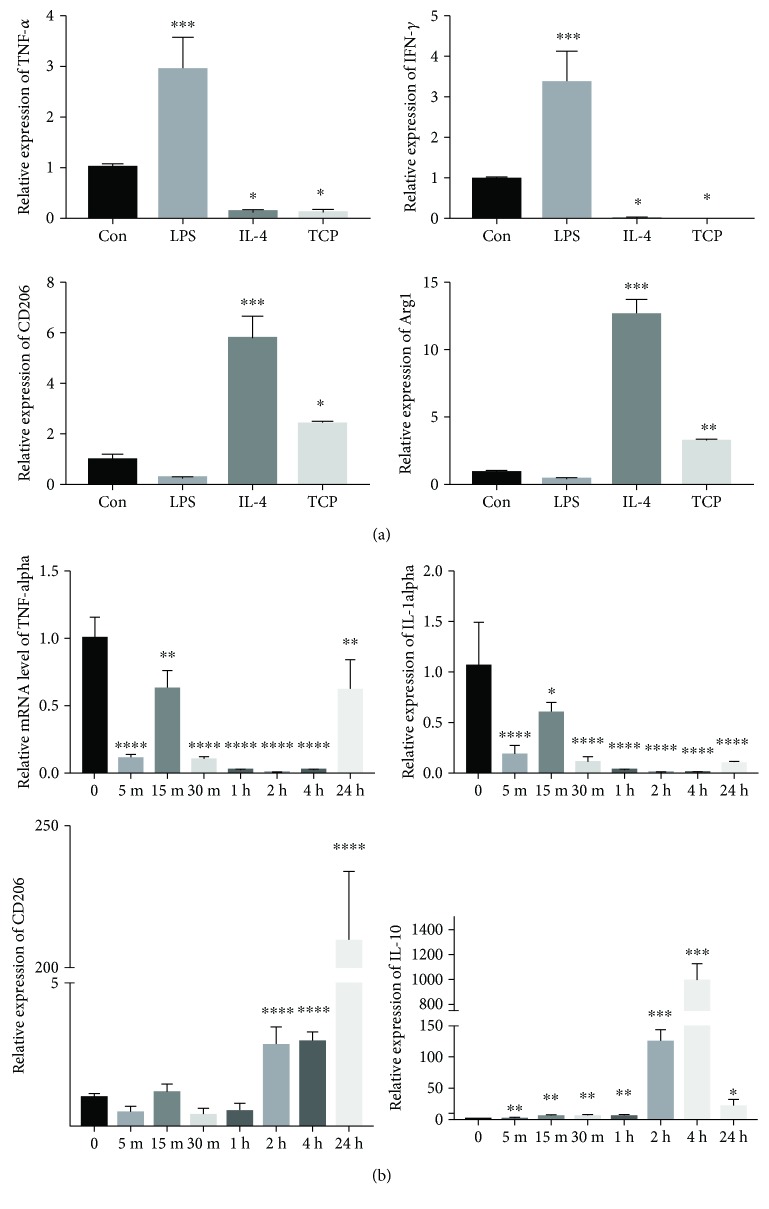
TCP facilitates M2 macrophage polarization. (a) Relative mRNA expression of TNF-*α*, IFN-*γ*, CD206, and ARG1 of Thp1 stimulated by control medium, LPS, IL-4, and TCP extract. (b) Relative mRNA expression of TNF-*α*, IL-1*α*, CD206, and IL-10 of Thp1 stimulated by TCP extract at different time points. ^∗^*p* < 0.05, ^∗∗^*p* < 0.01, ^∗∗∗^*p* < 0.001, and ^∗∗∗∗^*p* < 0.0001.

Previous studies have reported that TCP scaffolds can modify the immune microenvironment towards one that favours osteogenesis by switching the macrophage phenotype towards an M2 polarization [23]. Therefore, we decided to evaluate the anti-inflammatory ability of TCP through M1- (TNF-*α*, IFN-*γ*) and M2- (CD206, ARG1) associated markers and cytokines. Stimulation of Thp1 cells with TCP extract resulted in a shift towards the M2 phenotype with higher expression of M2 markers and lower expression of M1-associated cytokines (Figure 2(a)). The PCR results (Figure 2(b)) demonstrated that the most significant change in inflammatory gene expression occurred at 2 h, 4 h, and 24 h and therefore confirmed that changes occur rapidly when macrophages are in contact with TCP.

### 3.3. The Inhibition of EZH1 Caused by TCP Facilitates M2 Macrophage Polarization

**Figure 3:**
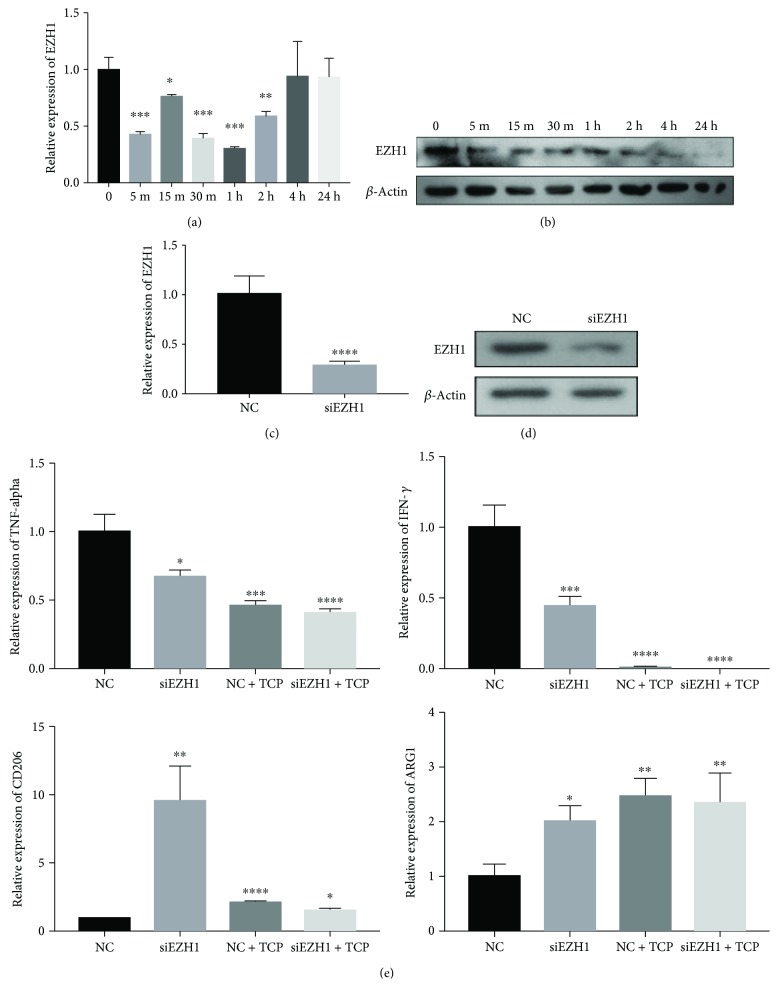
Inhibition of EZH1 caused by TCP stimulation facilitates M2 macrophage polarization. (a) Relative mRNA expression of EZH1 of Thp1 stimulated by TCP extract at different time points. (b) Western blot of EZH1 expression of Thp1 stimulated by TCP extract at different time points. (c) Relative mRNA expression of EZH1 of Thp1 cells after EZH1 knockdown. (d) Western blot of EZH1 expression of Thp1 cells after EZH1 knockdown. (e) Relative mRNA expression of TNF-*α*, IFN-*γ*, CD206, and ARG1 of Thp1 cells after EZH1 knockdown and stimulation by TCP extract. ^∗^*p* < 0.05, ^∗∗^*p* < 0.01, ^∗∗∗^*p* < 0.001, and ^∗∗∗∗^*p* < 0.0001.

There have been a large volume of published studies describing the role of methylase activity during macrophage polarization [15–17]. EZH1, part of a noncanonical PRC2 complex, specifically catalyzes H3K27 to prevent the derepression of PRC2 target genes. Further study showed that stimulation with TCP extracts significantly inhibited the gene expression and protein production of EZH1 when compared to the control group (Figures 3(a) and 3(b)).

In order to confirm the role of EZH1 during macrophage polarization, we silenced EZH1 in Thp1 cells with siRNA (Figures 3(c) and 3(d)). By analysing gene expression of macrophage polarization markers, it was found that siEZH1 significantly inhibited M2 macrophage polarization (Figure 3(e)).

### 3.4. The Inhibition of EZH1 Expression Is Associated with the Inhibition of the NF-*κ*B Pathway

**Figure 4:**
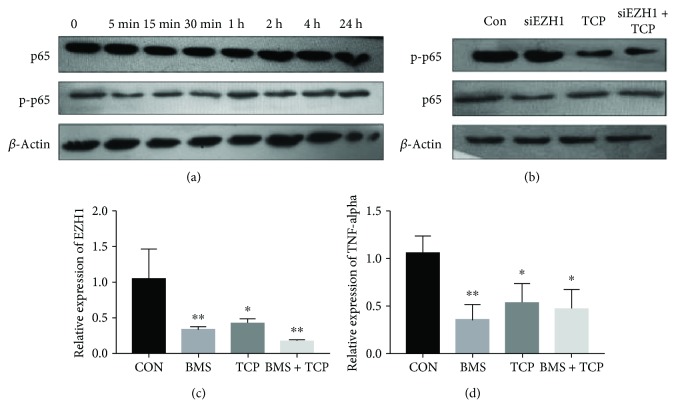
The inhibition of EZH1 expression is associated with the inhibition of the NF-*κ*B pathway. (a) Western blot of the NF-*κ*B signalling pathway of Thp1 stimulated by TCP extract at different time points. (b) Western blot of the NF-*κ*B signalling pathway of Thp1 cells after EZH1 knockdown and stimulation by TCP extract. (c) Relative mRNA expression of EZH1 of Thp1 cells after the inhibition of the NF-*κ*B pathway and stimulation by TCP extract. (d) Relative mRNA expression of TNF-*α*, IFN-*γ*, CD206, and ARG1 of Thp1 cells after the inhibition of the NF-*κ*B pathway and stimulation by TCP extract. ^∗^*p* < 0.05 and ^∗∗^*p* < 0.01.

Data from several studies have also identified a role of the NF-*κ*B pathway during macrophage polarization [1]. In the process of M1 polarization, the NF-*κ*B pathway is activated, which activates the transcription of M1-associated factors such as TNF-*α* and IFN-*γ*. Since the stimulation of TCP affects macrophages in less than 1 h, significant inhibition of p-p65 (one of the markers of NF-*κ*B pathway activation) was found (Figure 4(a)). However, after the silencing of EZH1, there was no significant difference between the siEZH1 group and the control group (Figure 4(b)). We therefore hypothesized that the NF-*κ*B pathway may be responsible for the inhibition of EZH1.

In order to determine the role of the NF-*κ*B pathway during EZH1 inhibition, an inhibitor for the NF-*κ*B pathway (BMS-345541) was utilized. It was found that it significantly affected the potency against IKK-1 and IKK-2 and subsequently inhibited the phosphorylation of p65. When treated with BMS-345541, the expression of EZH1 was significantly downregulated (Figure 4(c)), and since the NF-*κ*B pathway was inhibited, M2 macrophage polarization was upregulated (Figure 4(d)).

## 4. Discussion

The function of biological materials is associated with their mechanical and physicochemical properties [24]; however, their osteogenic effect is mainly dependent on their histocompatibility once implanted into host tissues. When a biomaterial is implanted, the body's immune system responds quickly, causing a brief, acute inflammatory response that leads to adaptive immunity [25]. The traditional view of the body's immune response is seen as a role of negative regulation to tissue repair function of biomaterials [26]. Recently, however, more studies have found the other sides of the immune regulation to tissue repair.

Macrophages are a group of well-known antigen-presenting cells. During tissue repair, they participate in almost every biological process after biomaterial implantation including the initial immune process, angiogenesis, mesenchymal stem cell recruitment, and differentiation [27–29]. Many in vivo studies have now shown that M1 macrophages dominate in the early process of tissue repair, while the M2 phenotypes are produced later during the process. Macrophages are however able to specifically polarize under various stimuli to either M1 (proinflammatory) or M2 (anti-inflammatory) phenotypes [29].

When it comes to biomaterials as TCP, cells derived from the monocyte/macrophage lineage is well known to be one of the first cell types contacting with biomaterials implanted [3]. As shown in Figure 1(b), here, they are able to specifically polarize under various stimuli to either M1 or M2 phenotypes or differentiate into osteoclasts or mutinucleated osteoclasts around TCP during TCP resorption [30]. There have been various previous papers that characterized these osteoclasts as foreign body giant cells which are associated with biomaterial rejection. Therefore, more recently, phenotypes of these osteoclasts have been associated with tissue regeneration and wound healing by researches demonstrating that even mutinucleated osteoclasts may be characterized as a tissue repair phenotype by demonstrating release of M2-related cytokines and growth factors [31].

In the present study, a positive role of macrophages in regulating bone repair was observed in response to TCP both *in vivo* and *in vitro*. Our results showed that macrophages gathered around TCP particles early during the bone repair. Once adhered, they express many surface molecules and cytokines that influences the surrounding microenvironment. To better understand the influence of TCP on macrophage polarization, we stimulated Thp1 cells with extracts from TCP *in vitro*. The result demonstrated a decreased expression of M1 marker genes (IFN-*γ*, TNF-*α*) while an increased expression of M2 markers (TGF-beta, VEGF) was observed.

Histone methylation plays a crucial role in macrophage polarization [32], but the epigenetic mechanism in the process mediated by TCP remains unclear. Previously, our research group found that EZH1 could regulate NF-*κ*B target genes through a nonclassical complex that was associated with SUZ12 and UTX. It was revealed that the polarization process of macrophages was mainly activated by the corresponding transcription factors through the NF-*κ*B and JAK-STAT pathways [1]. After activation, many transcription factors bind to promoter regions of M1 marker genes and begin their transcription [33]. In our study, we also observed that NF-*κ*B was repressed following stimulation with TCP extracts.

Classic NF-*κ*B consists of P50 and RelA (P65); the binding site of RelA 5′-AGAAATTCC-3′ was found in the promoter region of EZH1 gene sequence, but to date, there is no proof regarding the regulation of NF-*κ*B to EZH1 [34]. Therefore, we blocked NF-*κ*B with BMS-345541, an inhibitor of the NF-*κ*B pathway, and found that the expression of EZH1 decreased significantly with/without TCP stimulation. EZH1 belongs to the polycomb family, which can compensate the function of EZH1 in the PRC2 complex to catalyze H3K27me3 through the SET domain, leading to gene transcription inhibition. To investigate the role of EZH1 during macrophage polarization, we silenced EZH1 with siRNA. The expression of M1 markers including TNF-*α* and IFN-*γ* was both significantly decreased with or without TCP stimulation, which implied a positive role for EZH1 during the process of macrophages polarizing towards the M1 phenotype. We assume that there may be two potential mechanisms regarding the function of EZH1 as follows: (1) according to our previous study, EZH1 may interact with NF-*κ*B on the promoter regions of NF-*κ*B target genes, which is associated with M1 macrophage polarization and (2) as a marker for gene inhibition, EZH1 may target proximal sites on the promoter region of Tollip, a transcription factor that suppresses the NF-*κ*B pathway [35], which results in an enhancement of the NF-*κ*B pathway.

## 5. Conclusions

In the present study, it was found that TCP suppressed the activation of the NF-kappa B pathway and caused a decrease in EZH1 expression. The reduction of EZH1 impacted the downregulation of M1 marker genes, which resulted in a lower expression of M1 cytokines. These lower proinflammatory factors promoted a local microenvironment more favourable for bone regeneration. EZH1 seems to play an important role during the process of macrophage polarization and therefore might be an ideal target gene for future therapies aimed at modifying macrophage polarization following biomaterial implantation into host tissues.

## Figures and Tables

**Table 1:** The primer sequences used in RT-PCR.

Gene	Organism	Primer sequence
*IL-10*	*Homo sapiens*	Forward: 5′-GAGAAGCATGGCCCAGAAATC-3′ Reverse: 5′-GAGAAATCGATGACAGCGCC-3′
*IL-1α*	*Homo sapiens*	Forward: 5′-CTCCAGCTGGAGGAAGTTAAC-3′ Reverse: 5′-CTGACTCAAAGCTGGTGGTG-3′
*TNF-α*	*Homo sapiens*	Forward: 5′-CTGAACTTCGGGGTGATCGG-3′ Reverse: 5′-GGCTTGTCACTCGAATTTTGAGA-3′
*IFN-γ*	*Homo sapiens*	Forward: 5′-GGCCATCAGCAACAACATAAGC-3′ Reverse: 5′-GGGTTGTTGACCTCAAACTTGG-3′
*ARG1*	*Homo sapiens*	Forward: 5′-GCCCTTTGCTGACATCCTA-3′ Reverse: 5′-CACCAGGCTGATTCTTCCGT-3′
*CD206*	*Homo sapiens*	Forward: 5′-GGGTTGCTATCACTCTCTATGC-3′ Reverse: 5′-TTTCTTGTCTGTTGCCGTAGTT-3′
*RUNX2*	*Homo sapiens*	Forward: 5′-CTGTGGCATGCACTTTGACC-3′ Reverse: 5′-GACCCTGACTTTTCGGGGAG-3′
*OCN*	*Homo sapiens*	Forward: 5′-GCAAAGGTGCAGCCTTTGTG-3′ Reverse: 5′-GGCTCCCAGCCATTGATACAG-3′
*ALP*	*Homo sapiens*	Forward: 5′-TCAGAAGCTAACACCAACG-3′ Reverse: 5′-TTGTACGTCTTGGAGAGGGC-3′
*OSX*	*Homo sapiens*	Forward: 5′-CAACCTGCTAGAGATCTGAG-3′ Reverse: 5′-TGCAATAGGAGAGAGCGA-3′
*GAPDH*	*Homo sapiens*	Forward: 5′-TCAGCAATGCCTCCTGCAC-3′ Reverse: 5′-TCTGGGTGGCAGTGATGGC-3′

## Data Availability

The data used to support the findings of this study are included within the article.
